# Improvements in Compassion and Fears of Compassion throughout the COVID-19 Pandemic: A Multinational Study

**DOI:** 10.3390/ijerph20031845

**Published:** 2023-01-19

**Authors:** Marcela Matos, Kirsten McEwan, Martin Kanovský, Júlia Halamová, Stanley R. Steindl, Nuno Ferreira, Mariana Linharelhos, Daniel Rijo, Kenichi Asano, Sara P. Vilas, Margarita G. Márquez, Sónia Gregório, Gonzalo Brito-Pons, Paola Lucena-Santos, Margareth da Silva Oliveira, Erika Leonardo de Souza, Lorena Llobenes, Natali Gumiy, Maria Ileana Costa, Noor Habib, Reham Hakem, Hussain Khrad, Ahmad Alzahrani, Simone Cheli, Nicola Petrocchi, Elli Tholouli, Philia Issari, Gregoris Simos, Vibeke Lunding-Gregersen, Ask Elklit, Russell Kolts, Allison C. Kelly, Catherine Bortolon, Pascal Delamillieure, Marine Paucsik, Julia E. Wahl, Mariusz Zieba, Mateusz Zatorski, Tomasz Komendziński, Shuge Zhang, Jaskaran Basran, Antonios Kagialis, James Kirby, Paul Gilbert

**Affiliations:** 1 University of Coimbra, Center for Research in Neuropsychology and Cognitive Behavioral Intervention (CINEICC), 3004-531 Coimbra, Portugal; 2 Centre for Compassion Research and Training, College of Health, Psychology and Social Care, University of Derby, Derby DE22 1G, UK; 3 Institute of Social Anthropology, Faculty of Social and Economic Sciences, Comenius University, 814 99 Bratislava, Slovakia; 4 Institute of Applied Psychology, Faculty of Social and Economic Sciences, Comenius University, 814 99 Bratislava, Slovakia; 5 Compassionate Mind Research Group, School of Psychology, University of Queensland, Brisbane 4072, Australia; 6 Department of Social Sciences, University of Nicosia, Nicosia 2417, Cyprus; 7 Department of Psychological Counseling, Faculty of Psychology, Mejiro University, Tokyo 161-0032, Japan; 8 Behavior, Emotions, and Health Research Group, Department of Psychology, Faculty of Biomedical and Health Sciences, Universidad Europea de Madrid, 28670 Madrid, Spain; 9 Escuela de Psicología, Pontificia Universidad Católica de Chile, Santiago 8331150, Chile; 10 Evaluation and Treatment in Cognitive and Behavioral Psychotherapies—Research Group (GAAPCC), Pontifical Catholic University of Rio Grande do Sul, Porto Alegre 90619-900, Brazil; 11 Conectta: Mindfulness & Compassion, São Paulo 04038-001, Brazil; 12 Motivación Compasiva, Buenos Aires C1001, Argentina; 13 Neuroscience Department, Section of Psychiatry and Psychology, King Faisal Specialist Hospital and Research Centre (KFSH&RC), Jeddah 11564, Saudi Arabia; 14 School of Human Health Sciences, University of Florence, 50121 Florence, Italy; 15 Department of Economics and Social Sciences, John Cabot University, 00165 Rome, Italy; 16 Center for Qualitative Research in Psychology and Psychosocial Well-Being, National and Kapodistrian University of Athens, 157 72 Athens, Greece; 17 Department of Educational and Social Policy, University of Macedonia, 546 36 Thessaloniki, Greece; 18 Mindwork Psycological Center, 1620 Copenhagen, Denmark; 19 Department of Psychology, University of Southern Denmark, 5230 Odense, Denmark; 20 Department of Psychology, Eastern Washington University, Cheney, WA 99004, USA; 21 Department of Psychology, University of Waterloo, Waterloo, ON N2L 3G1, Canada; 22 Laboratoire Inter-Universitaire de Psychologie: Personnalité, Cognition et Changement Social, Grenoble Alpes University, 38400 Grenoble, France; 23 Centre Hospitalier Alpes Isère, C3R—Réhabilitation Psychosociale et Remédiation Cognitive, 38100 Grenoble, France; 24 CHU de Caen, Service de Psychiatrie Adulte, 14000 Caen, France; 25 UNICAEN, ISTS, GIP Cyceron, University of Normandy, 76000 Caen, France; 26 The Mind Institute Poland, 33-332 Warsaw, Poland; 27 Institute of Psychology, Department of General Psychology, SWPS University of Social Sciences and Humanities, 03-815 Warsaw, Poland; 28 Department of Cognitive Science, Nicolaus Copernicus University, 87-100 Torún, Poland; 29 Neurocognitive Laboratory, Centre for Modern Interdisciplinary Technologies, Nicolaus Copernicus University, 87-100 Torún, Poland; 30 School of Human Sciences, University of Derby, Derby DE22 1G, UK

**Keywords:** compassion, fears of compassion, longitudinal, multilevel modelling, multinational study, COVID-19, pandemic

## Abstract

During large-scale disasters, social support, caring behaviours, and compassion are shown to protect against poor mental health outcomes. This multi-national study aimed to assess the fluctuations in compassion over time during the COVID-19 pandemic. Respondents (Time 1 *n* = 4156, Time 2 *n* = 980, Time 3 *n* = 825) from 23 countries completed online self-report questionnaires measuring the flows of compassion (i.e., Compassionate Engagement and Action Scales) and fears of compassion toward self and others and from others (i.e., Fears of Compassion Scales) and mental health at three time-points during a 10-month period. The results for the flows of compassion showed that self-compassion increased at Time 3. Compassion for others increased at Time 2 and 3 for the general population, but in contrast, it decreased in health professionals, possibly linked to burnout. Compassion from others did not change in Time 2, but it did increase significantly in Time 3. For fears of compassion, fears of self-compassion reduced over time, fears of compassion for others showed more variation, reducing for the general public but increasing for health professionals, whilst fears of compassion from others did not change over time. Health professionals, those with compassion training, older adults, and women showed greater flows of compassion and lower fears of compassion compared with the general population, those without compassion training, younger adults, and men. These findings highlight that, in a period of shared suffering, people from multiple countries and nationalities show a cumulative improvement in compassion and reduction in fears of compassion, suggesting that, when there is intense suffering, people become more compassionate to self and others and less afraid of, and resistant to, compassion.

## 1. Introduction

With more than half a billion infections and over 6.5 million deaths worldwide and rising [1], the coronavirus (COVID-19) pandemic has proved to be a major and ongoing stressor. Several studies have shown a significant increase in psychological distress in the general population when compared to pre-pandemic levels [2,3,4,5], and these increases in psychological distress are likely to stem from COVID-related factors (e.g., fear of contracting the virus [6]) but also social factors such as isolation [7,8,9,10].

Self-isolation due to governmental measures (e.g., school/work closures, travel bans), had a significant impact on mental health [8,10,11,12,13], and it has been particularly taxing to those with pre-existing mental health conditions [3]. Self-isolation has also impacted social support in all three of its facets of received social support (e.g., “being able to receive help from others”), perceived social support (e.g., “experiencing connection with others”), and social embeddedness (e.g., “perceiving belonging to a community”) [14], with severe impairment felt due to physical distancing measures (e.g., inability to meet face-to-face, prohibitions on social gatherings). Social support has been a significant protective factor and predictor of better mental health outcomes in previous natural disaster settings [15,16,17].

Compassion is a process that has permeated the literature regarding the ability to engage in social support, social connection, and caring behaviours (for self and others) and is likely to be influential in the development of caring behaviours during the COVID-19 pandemic. Studies of compassion and social connection during the pandemic have indeed shown these to be protective factors against mental health [18,19]. The conceptualisation and definition of compassion researched and measured in this study derives from Compassion Focused Therapy (CFT), which is a Buddhist-informed [20] evolutionary and biopsychosocial approach, rooted in a broad range of sciences, including evolutionary psychology, attachment theory, developmental, clinical, and social psychology, neuroscience, and neurophysiology that was developed by Paul Gilbert for and with people with mental health difficulties, especially those who had problems with self-criticism, shame, and trauma and that often came from difficult backgrounds [21]. This approach indicates how the evolution of caring behaviour, primarily but not only parent-infant caring, created the motivation and appropriate psychophysiological infrastructures to detect and be sensitive to the needs and suffering of another (e.g., infant) and then act to alleviate distress and address needs. This definition highlights the stimulus-response algorithm of compassion motivation of (1) being prepared and willing to engage with (stimuli) indicators of distress and need (e.g., signals of distress such as crying) rather than ignore or avoid them, and (2) responding in appropriate (wise) ways to alleviate distress and address need [22]. Our capacities to engage and be sensitive to distress stimulate different psychophysiological processes to those of working out what to do and doing it [23]. Hence, there are very clear physiological effects of behaving compassionately that impact coping with challenges such as the COVID-19 pandemic. Reviews and meta-analyses of compassion-based interventions (such as CFT) demonstrate that interventions that help people become more empathic and sensitive to suffering and take an interest in being helpful mitigate mental health difficulties and improve wellbeing across both clinical and non-clinical populations [24,25,26,27].

Compassion can be given and received; hence, measures of compassion capture these different flows of having compassion for oneself, receiving compassion from others, and giving compassion to others. Interventions seeking to improve compassion in general community populations have been shown to improve compassion for self and from others [28,29,30] and, albeit to a lesser degree, compassion for others in certain contexts (i.e., teachers [31,32]). Life disruptors such as the COVID-19 pandemic have also complemented this pattern of findings, with compassion for self and from others (to a greater degree than compassion for others) acting as a buffer against poor mental health [19] and a facilitator of post-traumatic growth in this threatening context [18].

Cross-sectional data have shown that self-compassion and compassion from others may be protective factors for greater psychosocial wellbeing in the pandemic context [19,33,34,35,36]. However, changes in compassion across time have been scarcely explored and specifically, to our knowledge, how the flows of compassion (self-to-self, self-to-other, and other-to-self) fluctuate over the course of the COVID-19 pandemic is yet to be examined. The current longitudinal study offers an opportunity to examine compassion over time and in the context of a major life disrupting event (i.e., the pandemic).

In short-term studies investigating the temporal stability (i.e., re-test reliability) of compassion self-report measures, compassion towards oneself and others and received from others, have been found to be relatively stable over time [37,38,39]. In a longitudinal study across seven years, Lee et al. [40] found gender differences in the longitudinal trajectories of compassion towards others, with women having significantly higher levels than men throughout the follow-up period and across all age groups, although changes in compassion towards others were modest across age groups over a 7-year period. The trajectory of self-compassion revealed an inverse-U association with age and showed slight increases throughout the follow-up period, but it remained stable among participants in their 20s and 90s. Furthermore, this study revealed that increases in compassion towards others and self-compassion were associated with better mental well-being and lower loneliness over time across the adult lifespan [40].

Despite the apparent wellbeing benefits of compassion, there are instances where barriers and resistances to compassion (across the flows) can occur. These have been termed by Gilbert and colleagues as “fears of compassion” or fears, blocks and resistances (FBRs) to compassion [41], which can relate to early shame experiences and attachment trauma (e.g., where compassion triggers a grief response), valuing competitiveness (e.g., perceiving compassion as a barrier to success), or misconceptions around the term “compassion” (e.g., perceiving it as a low social rank position) [41,42]. FBRs to compassion are conceptualized as one of the key inhibitors of compassion across the three flows: self-compassion, compassion directed toward others, and compassion received from others [41]. Fears of compassion are related to the avoidance or fear response that arises when one seeks to develop or direct compassion and the perceived consequences of thinking, feeling, and behaving in compassionate ways, and they are linked, for example, to perceiving compassion as self-indulgent, self-pitying, weak, over-the-top, or too personally distressing. Blocks to compassion refer to situational factors, where someone may want to be compassionate but cannot do so because of a lack of opportunity, resources, information, availability, and so on. Resistances to compassion emerge when someone could be compassionate but chooses not to be because they believe it might be too costly for themselves or that there is no point in compassion [41,43]. Fears of compassion, especially for oneself and from others, have been consistently associated with mental health outcomes, such as depression, anxiety, stress, and well-being, and vulnerability factors, such as self-criticism and shame; these associations have been found to be even stronger in clinical populations [43].

In the pandemic context, cross-sectional data have shown that fears of compassion may be a risk factor for experiencing mental health difficulties (e.g., depression, anxiety, stress [44]; posttraumatic stress [19]). Moreover, fears of compassion were found to magnify the damaging impact of the COVID-19 pandemic on mental health and social safeness [44] and on posttraumatic stress [19] across 21 countries. Nevertheless, the way fears of compassion may change over time has never been examined and, specifically, how fears of compassion fluctuate across time during the COVID-19 pandemic. Again, the current longitudinal study offers a unique opportunity to examine this.

In addition, research exploring how specific sociodemographic variables are related to the flows of compassion and fears of compassion is lacking or has produced mixed results. Previous research has found that self-compassion is greater in men [45] and older adults [46], whilst compassion for others is greater in women [40,41]. There are no previous studies that directly compared compassion in healthcare workers vs. non-healthcare workers; however, prior to the pandemic, compassion fatigue in nurses was increasing gradually from 2010 to 2019, with the worst levels in Intensive Care Unit staff [47]. During the pandemic, lower levels of compassion satisfaction were detected in professionals working in areas with higher rates of contagion [48]. In terms of nationality demographics, a meta-analysis revealed the highest levels of compassion fatigue (and lowest levels of compassion satisfaction) were found in Asian Countries, with the opposite trend occurring in the Americas and Europe [47].

### Aims

The current study aimed to explore the natural fluctuation (time changes) of compassion (for self, for others, and from others) and of fears of compassion (for self, for others, and from others) across time during the COVID-19 pandemic in a multinational community sample. It was expected that compassion might increase whilst fears of compassion might decrease in correspondence with threatening events (peaks in COVID-19 cases and lockdown measures) due to increased opportunities to demonstrate or receive compassion in response to distress in self and others. This would be consistent within the context of historical large-scale crises, where social support has been found to be a primary coping-mechanism [49].

Furthermore, we aimed to examine whether specific variables (i.e., being a health professional, previous compassion training) would be associated with different baseline levels of the flows of compassion and fears of compassion, controlling for sociodemographic variables such as age and gender. It was hypothesized that being a health professional and having former compassion training would be associated with increased levels of compassion and decreased fears of compassion at the beginning of the pandemic.

## 2. Materials and Methods

### 2.1. Participants

The research sample was gathered from 23 different countries. At the first measurement, the total sample consisted of 4156 participants, mean age 41.91 (SD = 14.79) ranging from 18 to 91 years, with 80.73% (*N* = 3355) self-identified as women, 18.45% (*N* = 767) as men, 0.34% (*N* = 14) as other, and 0.48% (*N* = 20) preferred not to respond. The research sample comprised of 4156 participants from 23 countries: Argentine (ARG) *N* = 257, Australia (AUS) *N* = 109, Brazil (BRA) *N* = 406, Canada (CAN) *N* = 114, Chile (CHL) *N* = 282, China (CHN) *N* = 77, Columbia (COL) *N* = 50, Cyprus (CYP) *N* = 38, Denmark (DNK) *N* = 141, France (FRA) *N* = 115, Great Britain (GBR) *N* = 268, Greece (GRE) *N* = 145, Italy (ITA) *N* = 160, Japan (JPN) *N* = 522, Mexico (MEX) *N* = 181, Peru (*N* = 10), Poland (POL) *N* = 82, Portugal (PRT) *N* = 394, Saudi Arabia (SAU) *N* = 216, Slovakia (SVK) *N* = 46, Spain (ESP) *N* = 392, The United States of America (USA) *N* = 128, and Uruguay (*N* = 23). There were 1396 (33.6%) health professionals, and 2760 (66.4%) were not health professionals. A total of 1441 (34.7%) participants were involved in a compassion training and 2715 (65.3%) were not. At the second measurement, there were altogether 980 participants and at the third measurement 825 participants.

### 2.2. Measures

The online survey collected sociodemographic information (nationality, country of residence, age, gender) and administered self-report instruments assessing compassion (i.e., compassion for self, from others, for others) and fears of compassion (i.e., for self, from others, for others).

Compassionate Engagement and Action Scales (CEAS; Gilbert et al., 2017) include three scales that assess the three flows of compassion: self-compassion, compassion to others, and compassion received from others, with 13 items each. Each scale measures different elements of compassion Engagement (6 items and 2 filler items; e.g., “I am accepting, non-critical and non-judgmental of my feelings of distress”, “I notice and am sensitive to distress in others when it arises.”; “Other people are actively motivated to engage and work with my distress when it arises.”) and Action (4 items and 1 filler item; e.g., “I think about and come up with helpful ways to cope with my distress.”, “I take the actions and do the things that will be helpful to others”, “Others treat me with feelings of support, helpfulness and encouragement”). Participants are asked to rate each item on a ten-point Likert scale, based on how frequently it occurs, from 1 (never) to 10 (always). Each scale can be analysed in terms of the Engagement and Action components separately or as a single factor. Here we used each of the three flows of compassion as single factor scales. In the original study, the CEAS showed good internal consistencies and temporal reliability (Gilbert et al., 2017). In the present study, internal consistency ranged between good and excellent: Compassion for self: Engagement α = 0.74/Action α = 0.89; Compassion for others: Engagement α = 0.81/Action α = 0.88; Compassion from others: Engagement α = 0.91/Action α = 0.93.

Fears of Compassion Scales (FCS; Gilbert et al., 2011) are three scales that assess fears of compassion, one for each flow: (1) fears of feeling and expressing compassion for others (10-items; e.g., “People will take advantage of me if they see me as too compassionate”, “Being compassionate towards people who have done bad things is letting them off the hook”), (2) fears of receiving compassion from others (13-items; e.g., “Feelings of kindness from others are somehow frightening”, “I worry that people are only kind and compassionate if they want something from me”), and (3) fears of compassion for self (15-items; e.g., “I feel that I don’t deserve to be kind and forgiving to myself”, “I fear that if I become kinder and less self-critical to myself then my standards will drop”). Respondents were asked to rate on a five-point Likert scale how much they agree with each statement, from 0 (do not agree at all) to 4 (completely agree). Higher scores represent higher fears of compassion. In the original study, Cronbach’s alphas were 0.72 for FCS for others, 0.80 for FCS from others, and 0.83 for FCS self-compassion (Gilbert, et al., 2011). In the current study, internal consistencies ranged between 0.89 and 0.95 (FCS self-compassion α = 0.93, FCS compassion for others α = 0.89, FCS compassion from others α = 0.95).

### 2.3. Procedures

The current study is part of a broader longitudinal multinational study on compassion, social connectedness, and trauma resilience during the COVID-19 pandemic [19,32,44]. The study was approved by the Ethics Committee of the Faculty of Psychology and Educational Sciences of the University of Coimbra (UC; CEDI22.04.2020) and was conducted in compliance with the 1964 Helsinki Declaration and its later amendments. Local national ethical approval was also obtained whenever necessary. The current study had longitudinal design and the analysis used data collected in three time points over a 10-month period during the pandemic, across 23 countries from Europe, (United Kingdom, Portugal, Spain, Italy, France, Greece, Cyprus, Poland, Slovakia, Denmark), North America (USA, Canada), South America (Brazil, Argentina, Chile, Colombia, Mexico, Uruguay, Peru), Asia (China, Japan), Oceania (Australia), and the Middle East (Saudi Arabia). Participants recruited in Time 1 were asked to complete the self-report questionnaire survey at Time 2 and Time 3.

According to ourworldindata.org and covidtracker.bsg.ox.ac.uk, during Time 1 (mid-April 2020 to mid-May 2020), all countries had similar daily rates of confirmed new cases and deaths. With the notable exception of Japan (45%), the stringency index (a composite measure of nine of the response metrics including: school closures; workplace closures; the cancellation of public events; restrictions on public gatherings; closures of public transport; stay-at-home requirements; public information campaigns; restrictions on internal movements; and international travel controls) [50] varied roughly between 70–100% during this time period. At Time 2 (mid-September to mid-October 2020), all countries exhibited similar daily rates of new cases and deaths, with the exception of Argentina, where there was a spike in deaths in the first week of October. The stringency index during time 2 varied roughly between 30 and 70% in most countries, with the exception of Argentina, Chile, Colombia, China, and Australia, which still had higher levels of stringency. At time 3 (mid-January and mid-February 2021) all countries exhibited similar rates of new cases and deaths with the exception of the United Kingdom, Slovakia, Portugal, and Spain, which had elevated rates of these metrics compared to the remaining countries. During this same period, the stringency index was between 60 and 90% in most countries, with the exception of Australia, Japan, and Saudi Arabia, where stringency measures were lower. It is also important to note that, during Time 3, vaccination campaigns across all countries were underway, with most countries reporting less than 5% of the population as being vaccinated with the exceptions of the United Kingdom (22%), United States (13%), and Chile (11%).

The study was disseminated through social and traditional media platforms and institutional/professional emailing lists in each country, using snowball sampling. In addition, Facebook ads were used to promote participation among the general population in some countries. Prior to completing the online self-report questionnaires survey, participants were informed about the study aims and procedures and the voluntary and anonymous nature of participation. Confidentiality of the collected data was assured, and written informed consent was obtained in an online survey form before the completion of the study protocol. The online survey comprising the CEAS and the FCS scales was produced by the research team in English and translated to 11 other languages using forward/backward procedures by bilingual speakers to ensure the validity and language/cultural adaptation of the content. If the self-report questionnaire had already been validated for a particular language/country, then that version was used instead. The online surveys were hosted at the institutional account of the University of Coimbra in the online platform https://www.limesurvey.org/pt/ (accessed on 21 April 2020). The dissemination of the study across countries was supported by a website (https://www.fpce.uc.pt/covid19study/; accessed on 21 April 2020). The survey was self-paced and about 25 min long. There was no payment for completing the survey.

### 2.4. Data Analysis

To account for the cluster structure of data (three data points for each respondent, and respondents being nested within countries), multilevel models were chosen [51,52]. Each of the models had two levels: the respondents were the level 1 units, and the countries were the level 2 units.

The statistical procedure was as follows: (1) Fitting six multilevel models, with the same set of independent variables (predictors), but with a different dependent variable: (a) CEAS compassion for self; (b) CEAS compassion for others; (c) CEAS compassion from others; (d) FCS fear of compassion for self; (e) FCS fear of compassion for others; (f) FCS fear of compassion from others. (2) For each model, we tested the fit of several nested models with the data by likelihood-ratio tests and information criteria AIC (Akaike Information Criterion) and BIC (Bayes Schwarz Information Criterion) to obtain a final model with the best fit: (a) The null model included only the intercept. (b) The second model was the multilevel model, taking into account differences between countries (if adding countries as a random effect did not improve the fit, we could drop this level altogether). (c) The third model included main effects (predictors): time (factor with 3 levels), age (continuous), gender (factor with 2 levels), presence of a compassion training (factor with 2 levels), and the fact of being a health professional (factor with 2 levels). Adding these predictors should significantly improve the model, otherwise some or all of them could be dropped from the final model. (d) The fourth model included interaction effects: time with having a compassion training and time with being a health professional; these interactions allowed to compare different time effects between respondents who had compassion training (or who were health professionals) and the general population. (e) The fifth model included the autocorrelation effect; because each respondent provided three answers, residuals for each respondents could be autocorrelated with the result of s distortion of the model. (f) The sixth model was heteroscedastic; it estimated the different variance between strata (health professionals versus non-health professionals, compassion-trained versus compassion non-trained). Without taking into account the possible heteroscedasticity of the model, its estimations could be highly imprecise.

For statistical analyses, we used the R program version 4.0.3 [53] “nlme” package [54]. The effects were displayed through the “sjPlot” package [55]. As random effects, we used intercepts for participants and countries.

R^2^ (“variance explained”) statistics were used to measure the effect size of the model. However, there is no consensus as to the most appropriate definition of R^2^ statistics in relation to mixed-effect models [56,57,58,59]. Even though several methods for estimating the coefficient of determination (R^2^) for mixed-effect models are accessible, the estimation of R^2^ marginal and R^2^ conditional in the “MuMIn” package [60] was performed. The marginal R^2^ is the proportion of variability explained by the fixed effects/independent variable; the conditional R^2^ is the proportion of variability explained by both fixed and random effects (differences between respondents and differences between countries).

The likelihood-ratio tests and information criterium AIC (Akaike Information Criterion) for all models are presented in Supplementary Materials. It is evident from Tables S1–S6 in the Supplementary Materials that all multilevel models with country as the random effect consistently had a better fit than models that did not take differences between countries into account. Secondly, autocorrelation, heteroscedasticity, or both were present in all cases; therefore, fitting models that deal with these issues was appropriate and justified. An alpha level of 0.050 was used for all statistical analyses.

## 3. Results

Considering self-compassion (Table 1), there was no significant change between Time 1 and Time 2, but there was a significant increase in Time 3. Age and gender did not show any significant effects and neither did the comparison of health professionals and respondents with compassion training with the general population. There were no significant effects of interaction of sociodemographic variables.

Compassion for others (Table 2) showed significant increases at Time 2 and even more so in Time 3. Older respondents showed significantly less compassion for others than younger respondents, and women showed no significantly different effect in comparison to men. Health professionals and respondents with compassion training showed no significant difference in comparison to the general population. However, compassion for others among health professionals significantly decreased between Time 2 and Time 3, but there was no significant effect among respondents with compassion training.

Compassion from others did not change in Time 2, but it did increase significantly in Time 3 (Table 3). Older respondents showed no significant effect in comparison to younger respondents, and women showed no significantly different effect in comparison to men. Health professionals and respondents with compassion training showed no significant effects in comparison to the general population.

**Figure 1:**
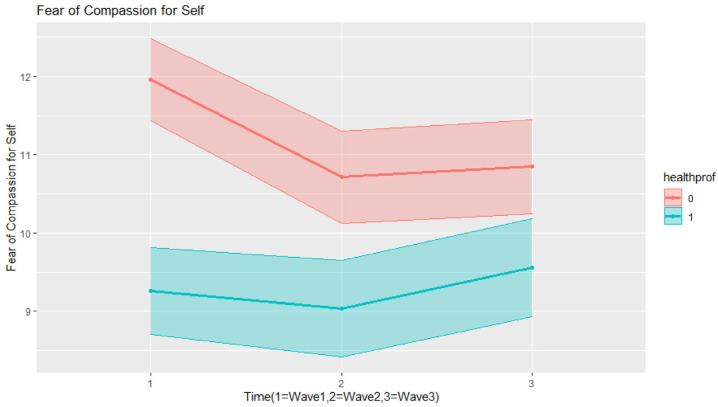
Interaction effects in the final model for fears of self-compassion.

Fears of self-compassion (Table 4) significantly decreased in Time 2, and this decrease was maintained at a similar level in Time 3. Older respondents showed significantly less fear of self-compassion than younger respondents, and so did women in comparison to men. Health professionals and respondents with compassion training had significantly less fear of self-compassion than the general population. Fear of self-compassion among health professionals significantly decreased over time, albeit these levels were low at Time 1 (baseline); hence, the magnitude of this decrease was smaller than the general population (as can be seen in Figure 1). Since our final model was heteroscedastic, we can report that variance among health professionals was 73% in comparison with the general population.

**Figure 2:**
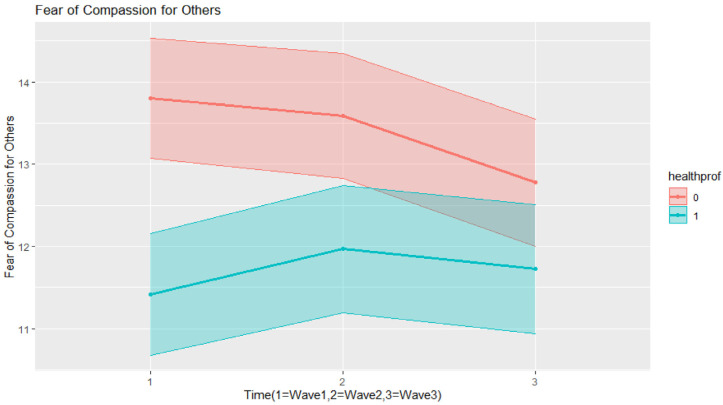
Interaction effects in the final model for fears of compassion for others.

Fear of compassion for others (Table 5) did not change in Time 2, but it did decrease significantly in Time 3. Older respondents showed significantly less fear of compassion for others than younger respondents, and so did women in comparison to men. Health professionals and respondents with compassion training had significantly less fear of compassion for others than the general population. As can be seen in Figure 2, fear of compassion for others among health professionals significantly decreased in time, although these fears were low at Time 1 (baseline), and they fluctuated with an increase in fears of compassion for others at Time 2 and a slight decrease at Time 3. Since our final model was heteroscedastic, we can report that variance among health professionals was 77% in comparison with the general population

We can see that fear of compassion from others (Table 6) did not show any significant change over time. Older respondents showed significantly less fear of compassion from others than younger respondents, and so did women in comparison to men. Health professionals and respondents with compassion training had significantly less fear of compassion from others than participants from the general population. Since there was no significant change over time, there were no significant effects on interaction.

## 4. Discussion

The current study examined the natural fluctuation of compassion and fears of compassion in a multinational community sample during the COVID-19 pandemic. Overall, the flows of compassion increased over time, whilst fears of compassion decreased during the pandemic. These results are consistent with previous findings from other major disasters, where social support was found to be linked with increased resilience and post-traumatic growth and emerged as a key factor for how people cope with adversity [61,62]. 

Specifically, for the whole sample, results revealed that self-compassion increased at Time 3, compassion for others increased at Time 2 and 3, and compassion received from others significantly increased at Time 3. While previous studies have not looked at changes in compassion over time in the long-term, short-term studies exploring the temporal stability of compassion self-report measures have documented that self-compassion, compassion for others, and compassion received from others seem to be relatively stable over time [37,38,39]. However, compassion is known to be malleable and with psychophysiological plasticity, meaning that compassion training can produce changes in the neural networks associated with threat processing, positive emotions, and emotion regulation [63,64,65,66] and can be improved with compassion-focused psychotherapeutic interventions, which also positively impact mental health [24,26]. Compassion emerged from the mammalian care-giving system algorithm for caring, where, if a stimulus indicates distress or need, then this activates behaviours to alleviate them [67]. Thus, in a time of elevated distress and shared human suffering (i.e., the pandemic), there may be more opportunities for individuals to be sensitive to and engage with suffering (in self and others) and to try to address that suffering with compassionate action. Thus, it seems that, during the pandemic, there was a natural tendency for individuals to become more able to engage with their own and other’s suffering and act in more compassionate ways towards themselves and others, while also becoming increasingly open to having compassion directed at them from other people.

In regard to inhibitors of compassion, fears of self-compassion reduced over time, fears of compassion for others significantly decreased in Time 3, whilst fears of receiving compassion from others did not significantly change over time. Notwithstanding the scarcity of previous studies examining changes in fears of compassion over time, intervention studies have documented that fears, blocks, and resistances to compassion decrease as a result of brief [30,42] and longer compassion-focused interventions (Irons and Maitland, 2020; Matos et al. 2022) and that these improvements are maintained over time [28,32]. These results suggest that, in the context of a major life disruptor event (i.e., the pandemic), inhibitors of compassion seem to diminish in the face of greater opportunities to express compassion.

When interpreting the results, it is important to note that baseline levels of compassion were already elevated in comparison to normative data prior to the pandemic [37], whereas levels of fears of compassion were lower at baseline [41]. Despite these higher levels of compassion at baseline, participants still showed significant increases across time; despite lower fears of compassion at baseline, these still tended to further decrease across the pandemic. This provides evidence of a cumulative improvement in compassion and reduction in fears of compassion over the course of the pandemic and suggests that, when there is intense suffering, people seem to become more compassionate and less afraid of and resistant to compassion. This might also be related to the specificity of the pandemic threat, which, such as other large-scale tragedies, seems to activate a compassionate motivation to care for others and for oneself. At the same time, the public messages at the beginning of the pandemic were very focused on caring and protecting others and oneself.

In terms of sociodemographic influences, in self-compassion and compassion from others, there were no differences in any sociodemographic variables. Sociodemographic influences were found in terms of compassion for others, which was found to increase in the general population, but in contrast, it decreased in health professionals between Time 2 and Time 3. This could potentially be related to elevated burnout and compassionate fatigue in healthcare workers as the pandemic continued [68,69]. It is interesting that this was not true for those who were trained in compassion, who may therefore be more resilient to burnout and compassion fatigue [70]. Compassion for others was also lower amongst older participants, which might be related to having greater vulnerability to COVID-19 and a higher threat perception towards others during this period, which thus could reduce their motivation to be compassionate towards others. Furthermore, women revealed no significantly different effect in compassion for others in comparison to men. This is in contrast with a prior longitudinal study showing that women had significantly higher levels of compassion towards others than men across seven years and age groups [40].

In terms of sociodemographic influences on fears of compassion, across all the flows of fears of compassion (i.e., for self, from others, and for others), health professionals, respondents with compassion training, older respondents, and women had significantly less fear of self-compassion than the general population. Several studies have found that health professionals engaging with compassion training showed reduced fears of compassion [71,72]. The finding that older respondents and women had fewer fears of compassion is consistent with clinician observations of patients undergoing compassion focused therapies [73].

To summarize, there was no influence of sociodemographics on compassion, apart from healthcare professionals and older adults showing reductions in compassion for others as the pandemic progressed. Fears of compassion were lower in healthcare professionals, those with compassion training, older adults, and women.

### Limitations and Future Directions

A limitation of the current study pertains to the dropout rate across time. While dropouts are to be expected in a study with a longitudinal design and where the multiple measurements generally coincide with peaks in pandemic cases and associated lockdown measures, this raises the question of whether there were differences between participants who dropped out from those who did not. For example, it may be that participants who remained in the study were those more prone to be compassionate and to be less afraid of compassion. In the future, research could explore differences between these participants in baseline levels of compassion and fears of compassion and also in indicators of psychological distress, as these may influence the activation of compassionate and caring motivational systems. Furthermore, there was an uneven gender distribution in this study, with more respondents identifying as women. Although no gender differences have been reported in the self-compassion and compassion from other scales, women have been found to score higher than men in compassion towards others [40,41]. Thus, future studies should seek to recruit more gender-balanced samples. Another limitation pertains to the non-probabilistic sampling method used, which may affect the extrapolation of the findings to the whole population.

Differences across the 21 countries in terms of rates of COVID-19 and the timing of peaks of infection and associated lockdown measures may have impacted the levels of compassion and fears of compassion. Additionally, it is possible that cross-cultural differences in the compassion flows, which have been reported in previous studies [74] and could be related, for example, to diverse perceptions of the meaning of compassion among countries/cultures [75], and the type of strategies implemented by different countries to limit the spread of the virus across the of the pandemic waves might influence the fluctuation in the flows of compassion and fears of compassion. It is therefore important that reliable compassion measures that are validated multi-nationally are adopted in future research. Nevertheless, a key strength of the current study was the multivariate multilevel methodology used and the consistency of the effects across all 21 countries, thus supporting the validity of the measures used and the universality of the cumulative improvement in compassion and fears of compassion over the course of the pandemic.

In light of the current findings, it would be pertinent for future research to explore other variables that might play a role in explaining the documented increases in the flows of compassion and decreases in fears of compassion across time and to map how these changes relate to changes in other variables related to the perceived threat of COVID-19, psychological distress, trauma, and social connection, for example. In fact, this study is part of an ongoing broader multinational project that aims to prospectively investigate the buffering effects of compassion and fears of compassion throughout the pandemic. 

## 5. Conclusions

This study assessed the natural fluctuation of compassion and fears of compassion in a multinational community sample across 10 months during the pandemic. Compassion increased while fears of compassion decreased during the pandemic, which are consistent with previous findings from major disasters, where forms of social support become a main resource for coping. It is likely that the pandemic, a time of elevated distress and shared human suffering, provided more opportunities for people to respond to distress with compassion. In addition, during the pandemic, messages received from governments and public health organizations pertained to caring for each other. Sociodemographic variables influenced these fluctuations in compassion, with healthcare professionals and older adults showing less compassion for others as the pandemic progressed, possibly due to burnout or increased vulnerability to contagion, while in terms of fears of compassion, fears were lower in healthcare professionals, those with compassion training, older adults, and women. Compassion is known to have plasticity and can be trained; hence, engaging with compassion-focused interventions could offer a resource for coping during large-scale uncontrollable events. This could be particularly relevant for healthcare professionals as a way of promoting emotional regulation and compassionate skills whilst reducing burnout and compassionate fatigue, especially in the face of extended major threatening events, such as the pandemic.

## Figures and Tables

**Table 1:** Estimates of the final model for self-compassion.

Fixed Effects	β (SE)	*p*-Value	
Intercept	68.03 (0.85)	0.0000	
Time 2	−0.25 (0.17)	0.1541	
Time 3	0.93 (0.20)	0.0000	
Age	0.02 (0.01)	0.1476	
Gender	0.65 (0.49)	0.1814	
Health professionals	0.72 (0.42)	0.0888	
Compassion training	0.42 (0.42)	0.3243	
**Variance components**	**σ^2^**	**Effect size**	**Autocorrelation**
Respondents	102.01		
Countries	2.56		
Residuals	86.49		
φ			0.28
R^2^		0.35	

**Table 2:** Estimates of the final model for compassion for others.

Fixed Effects	β (SE)	*p*-Value	
Intercept	76.94 (0.98)	0.0000	
Time 2	0.49 (0.20)	0.0156	
Time 3	1.30 (0.23)	0.0000	
Age	−0.03 (0.01)	0.0432	
Gender	0.82 (0.44)	0.0637	
Health professionals	0.80 (0.43)	0.0693	
Compassion training	0.57 (0.43)	0.1872	
Time 2: health professionals	−0.06 (0.33)	0.8644	
Time 3: health professionals	−0.76 (0.38)	0.0463	
Time 2: compassion training	−0.61 (0.33)	0.0617	
Time 3: compassion training	−0.52 (0.38)	0.1696	
intercept	76.94 (0.98)	0.0000	
**Variance components**	**σ^2^**	**Effect size**	**Autocorrelation**
Respondents	81		
Countries	9.8		
Residuals	70.06		
Φ			0.34
R^2^		0.40	

**Table 3:** Estimates of the final model for compassion from others.

Fixed Effects	β (SE)	*p*-Value	
Intercept	61.76 (1.13)	0.0000	
Time 2	0.30 (0.23)	0.1916	
Time 3	0.75 (0.26)	0.0040	
Age	−0.00 (0.02)	0.8553	
Gender	−1.18 (0.61)	0.0523	
Health professionals	0.98 (0.53)	0.0620	
Compassion training	0.37 (0.53)	0.4851	
**Variance components**	**σ^2^**	**Effect size**	**Autocorrelation**
Respondents	152.52		
Countries	6.86		
Residuals	146.41		
φ			0.24
R^2^		0.31	

**Table 4:** Estimates of the final model for fears of self-compassion.

Fixed Effects	β (SE)	*p*-Value	
Intercept	20.66 (0.80)	0.0000	
Time 2	−1.24 (0.32)	0.0001	
Time 3	−1.11 (0.34)	0.0012	
Age	−0.14 (0.01)	0.0000	
Gender	−1.62 (0.40)	0.0001	
Health professionals	−2.70 (0.35)	0.0000	
Compassion training	−3.82 (0.34)	0.0000	
Time 2: health professionals	1.01 (0.45)	0.0238	
Time 3: health professionals	1.41 (0.48)	0.0035	
**Variance components**	**σ^2^**	**Effect size**	**Autocorrelation**
Respondents	68.89		
Countries	4.93		
Residuals	37.09		
R^2^		0.33	

**Table 5:** Estimates of the final model for fears of compassion for others.

Fixed Effects	β (SE)	*p*-Value	
Intercept	18.56 (0.85)	0.0000	
Time 2	−0.21 (0.24)	0.3791	
Time 3	−1.02 (0.28)	0.0003	
Age	−0.05 (0.01)	0.0000	
Gender	−1.52 (0.29)	0.0000	
Health professionals	−2.39 (0.25)	0.0000	
Compassion training	−3.94 (0.24)	0.0000	
Time 2: health professionals	0.76 (0.35)	0.0285	
Time 3: health professionals	1.33 (0.41)	0.0011	
**Variance components**	**σ^2^**	**Effect size**	**Autocorrelation**
Respondents	18.75		
Countries	11.02		
Residuals	29.16		
φ			0.40
R^2^		0.34	

**Table 6:** Estimates of the final model for fear of compassion for others.

Fixed Effects	β (SE)	*p*-Value	
Intercept	19.98 (0.77)	0.0000	
Time 2	−0.25 (0.21)	0.2154	
Time 3	−0.37 (0.22)	0.0915	
Age	−0.13 (0.01)	0.0000	
Gender	−1.24 (0.35)	0.0004	
Health professionals	−2.12 (0.30)	0.0000	
Compassion training	−2.00 (0.30)	0.0000	
**Variance components**	**σ^2^**	**Effect size**	**Autocorrelation**
Respondents	49.98		
Countries	6.25		
Residuals	30.25		
R^2^		0.33	

## Data Availability

Data cannot be shared publicly because it is part of an ongoing longitudinal study. Data are available from the University of Coimbra Institutional Data Access (contact via cineicc@fpce.uc.pt) for researchers who meet the criteria for access to confidential data after the completion of the longitudinal study.

## Supplementary Materials

The following supporting information can be downloaded at: https://www.mdpi.com/article/10.3390/ijerph20031845/s1.
