# Supplementary material for: Improvements in Compassion and Fears of Compassion throughout the COVID-19 Pandemic: A Multinational Study
Source: Int J Environ Res Public Health. 2023 Jan 19;20(3):1845. doi: 10.3390/ijerph20031845 (PMC9915071; doi:10.3390/ijerph20031845)
Supplement: Supplementary file 1 [file ijerph-20-01845-s001.zip › ijerph-2156379-supplementary.pdf]

International Journal of Environmental Research and Public Health

Supplementary Materials

Table S1. Compassion for self.

Model	Deviance	χ^2 (df)	p-Value	AIC	Autocor	Variance	Multilevel
m0	94910	-	-	94915	No	homoscedastic	no
m1	94856	54 (1)	< .001	94863	No	homoscedastic	yes
m2	94800	56 (6)	< .001	94819	No	homoscedastic	yes
m3	94798	2 (1)	n.s	94820	No	homoscedastic	yes
m4 *	94797	3 (1)	n.s	94819	No	homoscedastic	yes
**m5**	**94672**	**135 (1)**	**< .001**	**94694**	**Yes**	**homoscedastic**	**yes**

Note. χ^2 = chi-square, df = degrees of freedom, AIC = Akaike information Criterion, autocor = autocorrelation, *m4 is compared with m2.

Table S2. Compassion for others.

Model	Deviance	χ^2 (df)	p-value	AIC	Autocor	Variance	Multilevel
m0	92018	-	-	92024	No	homoscedastic	no
m1	91753	265 (1)	< .001	91761	No	homoscedastic	yes
m2	91711	42 (6)	< .001	91733	No	homoscedastic	yes
m3	91703	8 (1)	< .010	91727	No	homoscedastic	yes
m4*	91705	6 (1)	< .050	91731	No	homoscedastic	yes
**m5**	**91522**	**193 (1)**	**< .001**	**91549**	**Yes**	**homoscedastic**	**yes**
m6	91518	4 (3)	n.s	91552	Yes	heteroscedastic	yes

Note. χ^2 = chi-square, df = degrees of freedom, AIC = Akaike information Criterion, autocor = autocorrelation, *m4 is compared with m2.

Table S3. Compassion from others.

Model	Deviance	χ^2 (df)	p-Value	AIC	Autocor	Variance	Multilevel
m0	101438	-	-	101444	no	homoscedastic	no
m1	101371	67 (1)	< .001	101379	no	homoscedastic	yes
m2	101353	18 (6)	< .010	101374	no	homoscedastic	yes
m3	101349	4 (1)	n.s	101374	no	homoscedastic	yes
m4*	101350	3 (1)	n.s	101375	no	homoscedastic	yes
**m5**	**101255**	**98 (1)**	**< .001**	**101284**	**yes**	**homoscedastic**	**yes**
m6	101254	1 (1)	n.s	101289	yes	heteroscedastic	yes

Note. χ^2 = chi-square, df = degrees of freedom, AIC = Akaike information Criterion, autocor = autocorrelation, *m4 is compared with m2.

Table S4. Feasr of self-compassion.

model	deviance	χ^2 (df)	*p*-value	AIC	Autocor	variance	multilevel
m0	43810	-	-	43816	no	homoscedastic	no
m1	43288	522 (1)	< .001	43295	no	homoscedastic	yes
m2	42868	420 (6)	< .001	42887	no	homoscedastic	yes
m3	42859	9 (1)	< .050	42882	no	homoscedastic	yes
m4*	42868	1 (1)	n.s.	42889	no	homoscedastic	yes
m5	42865	3 (1)	n.s.	42890	yes	homoscedastic	yes
**m6**	**42786**	**81 (1)**	**< .001**	**42813**	**no**	**heteroscedastic**	**yes**

Note. χ^2 = chi-square, df = degrees of freedom, AIC = Akaike information Criterion, autocor = autocorrelation, *m4 is compared with m2.

Table S5. Fears of compassion for others.

Model	Deviance	χ^2 (df)	p-Value	AIC	Autocor	Variance	Multilevel
m0	36316	-	-	36322	No	homoscedastic	no
m1	35106	1210 (1)	< .001	35115	No	homoscedastic	yes
m2	34605	501 (6)	< .001	34628	No	homoscedastic	yes
m3	34594	11 (1)	< .01	34620	No	homoscedastic	yes
m4*	34596	8 (1)	< .05	34624	No	homoscedastic	yes
m5	34592	4 (1)	< .05	34621	yes	homoscedastic	yes
**m6**	**34532**	**60 (3)**	**< .001**	**34568**	**yes**	**heteroscedastic**	**yes**

Note. χ^2 = chi-square, df = degrees of freedom, AIC = Akaike information Criterion, autocor = autocorrelation, *m4 is compared with m2.

Table S6. Fears of compassion from others.

Model	Deviance	χ^2 (df)	p-Value	AIC	Autocor	Variance	Multilevel
m0	42330	-	-	42335	No	homoscedastic	no
m1	41704	626 (1)	< .001	41711	No	homoscedastic	yes
m2	41393	311 (6)	< .001	41412	No	homoscedastic	yes
m3	41392	1 (1)	n.s.	41415	No	homoscedastic	yes
m4*	41392	1 (1)	n.s	41416	No	homoscedastic	yes
m5	41390	2 (1)	n.s	41423	Yes	homoscedastic	yes
**m6**	**41330**	**72 (3)**	**< .001**	**41356**	**No**	**heteroscedastic**	**yes**

Note. χ^2 = chi-square, df = degrees of freedom, AIC = Akaike information Criterion, autocor = autocorrelation, *m4 is compared with m2.
